# Satisfied employees, stronger teams: linking need satisfaction to team-boosting behaviours via work engagement

**DOI:** 10.3389/fpsyg.2026.1917631

**Published:** 2026-09-23

**Authors:** Vallenice Van Blerk, Leon T. De Beer

**Affiliations:** 1 WorkWell Research Unit, Faculty of Economic and Management Sciences, North-West University, Potchefstroom, South Africa; 2 Department of Psychology, Faculty of Social and Educational Sciences, Norwegian University of Science and Technology, Trondheim, Norway

**Keywords:** basic psychological need satisfaction, bifactor exploratory structural equation modelling, self-determination theory, team-boosting behaviours, work engagement

## Abstract

**Purpose:**

Modern work teams are increasingly diverse, dynamic, and dispersed. For organisations to thrive in this context, they must prioritise team functioning. Individual team members play a central role in shaping team climate, dynamics, and performance. This study investigates relationships between basic psychological need satisfaction (BPNS), work engagement, and team-boosting behaviours (TBB). Drawing on self-determination theory, it examines whether employees’ need satisfaction relates to discretionary behaviours that enhance team climate through work engagement, linking individual motivation to team-strengthening outcomes.

**Design/methodology/approach:**

A cross-sectional survey design was employed with employees (*n* = 332). Participants completed validated self-report measures of BPNS, work engagement, and TBB. Structural equation modelling using a bifactor exploratory structural equation modelling framework tested relationships, isolating global and specific need satisfaction factors.

**Findings:**

Global BPNS positively predicted work engagement and all team-boosting behaviours (mood-enhancing, energising, unifying). Work engagement mediated between global need satisfaction and each team-boosting dimension. Specific competence satisfaction predicted all TBB, while relatedness satisfaction predicted only mood-enhancing behaviours. Effects were primarily driven by global BPNS.

**Practical implications:**

Organisations should prioritise need-supportive environments that foster overall psychological need satisfaction, which may in turn relate to greater engagement and more frequent team-directed discretionary behaviours. Interventions should focus on autonomy-supportive leadership, competence development through feedback and mastery experiences, and relatedness support through psychological safety and connection-building practices.

**Originality/value:**

This study uniquely integrates BPNS, work engagement, and team-boosting behaviours within a single model and suggests how individual motivational experiences may relate to team-directed behaviours, consistent with a bottom-up pathway to team climate enhancement.

## 1 Introduction

As organisational structures evolve towards flatter models and work environments become increasingly complex and interdependent, the nature of work teams and their functioning is transforming (Adekoya et al., 2022; Gagné et al., 2022). Digital platforms also render contemporary work teams more diverse and more likely to operate across locations (Stratone et al., 2022). This dynamism has contributed to workplaces in which teams often function with minimal managerial oversight (Jungert et al., 2018). Accordingly, it is important to extend our focus beyond management and also consider how collegial interactions shape positive individual, team, and organisational outcomes.

Self-determination theory (SDT) offers a valuable framework by highlighting motivational processes that are shaped by employees’ experiences of psychological need satisfaction (Deci and Ryan, 2000, 2008; Gopalan et al., 2017; Ryan and Deci, 2020; Ryan et al., 2021). The three fundamental psychological needs identified by SDT—autonomy, competence, and relatedness—are essential for fostering positive behaviours, shaping motivation, and directing cognition (Autin et al., 2022; Van den Broeck et al., 2016; Wang et al., 2021). When employees experience satisfaction of these basic psychological needs (BPN), they are more likely to engage more fully with their work and colleagues. Such engagement reflects increased energy and purpose in work-related tasks and, in turn, is associated with higher levels of work engagement (Heyns et al., 2022; Mokgata et al., 2023; Rahmadani et al., 2019; Van Wingerden et al., 2018). As Bakker et al. (2023) explain, work engagement is a work-related state characterised by vigour, dedication, and absorption in work tasks.

Prior research has demonstrated the influence of work engagement on a range of organisational outcomes, including employee wellbeing, proactive behaviours, organisational commitment, organisational citizenship behaviours, and performance (Bakker, 2017; Bakker and Albrecht, 2018; Costa et al., 2014). Employees’ needs, motivation, and behaviour may therefore have implications not only for individual performance but also for team functioning and broader organisational culture.

Recently, team-boosting behaviours (TBB) were introduced to capture how individual employee behaviours can shape the overall team climate (Fortuin et al., 2021). TBB include mood-enhancing (e.g., breaking negative team atmospheres with humour), energising (e.g., stimulating the team), and unifying behaviours (e.g., strengthening bonds between members), all of which can influence team functioning (Fortuin et al., 2021). These behaviours sit within a broader literature on group affect, which shows that moods and emotions converge in teams through interpersonal processes and shape team outcomes (Barsade & Knight, 2015). Building on this work, the present study examines whether employees’ BPN satisfaction relates to behaviours that contribute to a positive team climate via work engagement.

Previous studies have mainly focused on environments supporting employees’ BPN satisfaction (Gillet et al., 2012; Gillet et al., 2020; Jungert et al., 2018; Rigby and Ryan, 2018; Ryan et al., 2022). In contrast, this study focuses on how employees whose needs are satisfied may enact behaviours that may enhance team climate. This approach builds on emotional contagion theory, which suggests that affective states and related behaviours can spread through interpersonal interaction, potentially shaping the team’s shared mood (Hernandez Grande et al., 2025; Rhee et al., 2020). The study also draws on broaden-and-build theory, which proposes that positive affect broadens thought–action repertoires and helps individuals build enduring personal resources (Diener et al., 2020; Fredrickson, 2013). In combination, these perspectives suggest that basic psychological need satisfaction (BPNS) may be associated with positive affect and engagement that support mood-enhancing, energising, and unifying behaviours directed at the team.

Against this background, the specific gap addressed in this study can be stated directly. Research on TBB is still emerging and has concentrated on the consequences of these behaviours for team processes, whereas their motivational antecedents remain largely unexamined (Fortuin et al., 2021; Fortuin et al., 2023). Furthermore, although BPNS is a well-established correlate of work engagement, it is unclear whether, and through which motivational state, individual need satisfaction relates to discretionary behaviours that are directed at the team, not at the task or the organisation. The present study addresses this gap by testing work engagement as an intervening variable between employees’ need satisfaction and the three TBB dimensions, thereby connecting individual-level motivational experiences to behaviours that are theorised to feed into team climate. Understanding how employees’ BPN satisfaction relates to work engagement and positive team behaviours may inform efforts to strengthen employee motivation and team functioning. Accordingly, this study investigates the direct and indirect relations among BPN satisfaction, work engagement and TBB in a diverse sample of employees.

## 2 Literature review

### 2.1 Concept overview and hypothesis development

Self-determination theory (SDT) explains motivational quality by proposing that employees function most optimally when BPN for autonomy, competence, and relatedness are satisfied (Deci and Ryan, 2000; Ryan and Deci, 2020; Vansteenkiste et al., 2020). In organisational contexts, these needs are theorised to be essential and directional: when satisfied, they energise and guide cognition and behaviour toward self-endorsed action, whereas need deprivation or thwarting is linked to maladaptive functioning (Chen et al., 2015; Ryan and Deci, 2017; Tóth-Király et al., 2022; Vansteenkiste and Ryan, 2013). Consistent with this view, BPN satisfaction has been associated with wellbeing, resilience to stressors, and motivationally adaptive work outcomes (Breaugh, 2021; Cantarero et al., 2021; Vansteenkiste et al., 2020). At the same time, basic psychological needs theory treats the three-need set as well supported but not closed, and candidate needs such as beneficence (Martela and Ryan, 2016) and novelty (González-Cutre et al., 2016) remain the subject of ongoing evaluation (Vansteenkiste et al., 2020; Vansteenkiste et al., 2023). In this study, the term need satisfaction therefore refers to SDT-defined need satisfaction, operationalised through autonomy, competence, and relatedness.

One particularly relevant motivational outcome is work engagement, described as a fulfilling work-related state characterised by vigour, dedication, and absorption (Bakker et al., 2023). Work engagement is often situated within Job Demands–Resources (JD–R) theory, which proposes that resources help employees meet demands and foster a motivational process culminating in engagement (Bakker et al., 2023; Bakker and Albrecht, 2018). However, SDT helps specify why resources are motivating by clarifying the psychological experiences (i.e., need satisfaction) through which supportive conditions translate into engagement (Breaugh, 2021; Morin et al., 2023). Similarly, engagement itself has been conceptualised as an expression of underlying human motivation and need fulfilment, which positions need satisfaction as the more fundamental construct in this sequence (Pincus, 2023). Empirically, BPNS has been consistently associated with work engagement across contexts (e.g., Heyns et al., 2022; Rahmadani et al., 2019; Vansteenkiste et al., 2020). Accordingly, the present study proposes:

*H1*: *BPNS is positively related to work engagement*.

Beyond individual functioning, contemporary team-based work also depends on how employees shape the team’s socio-emotional climate through everyday interaction. Team-boosting behaviours (TBB) were recently introduced to capture team-directed discretionary behaviours aimed at improving the team’s affective and relational conditions (Fortuin et al., 2021). Unlike broader extra-role or organisational citizenship behaviours, TBB are defined by their explicit target: the team’s mood, energy, and cohesion, rather than general helpfulness or discretionary performance (Fortuin et al., 2021; Fortuin et al., 2023). Fortuin et al. (2021) distinguish three forms: mood-enhancing behaviours (e.g., using humour or reframing to lift the team atmosphere), energising behaviours (e.g., initiating activities that stimulate collaboration and momentum), and unifying behaviours (e.g., fostering inclusive connection and strengthening bonds). Notably, initial evidence suggests that while a designated “team booster” can be beneficial, spontaneous TBB enacted by team members may show stronger associations with team processes such as team engagement and team climate (Fortuin et al., 2021). This positions TBB as a promising bottom-up mechanism through which individual motivational experiences may accumulate into team-level functioning. TBB can also be situated in relation to work on team emotional intelligence, which describes the group-level norms through which teams manage members’ emotions and needs (Druskat & Wolff, 2001); the emotion-management and relationship-building norms described there overlap conceptually with the mood-enhancing and unifying TBB dimensions, although TBB are individual behaviours, not shared norms.

From an SDT perspective, BPNS should increase the likelihood of interpersonal behaviours that build and maintain positive relationships and constructive interaction patterns. Relatedness satisfaction, in particular, may support connection and goodwill toward colleagues, which can translate into unifying and mood-enhancing behaviours, especially during challenges (Chen et al., 2015; Fortuin et al., 2021). Autonomy and competence satisfaction may similarly support volitional and efficacious action, making it more likely that employees initiate energising behaviours that mobilise the team (Fortuin et al., 2021). This aligns with evidence that need-satisfied employees are more inclined toward supportive, prosocial, and positive interpersonal behaviour (Gillet et al., 2020). Because the bifactor-ESEM operationalisation used here separates a global need satisfaction factor from specific autonomy, competence, and relatedness factors, the analyses could also examine whether each specific need relates to TBB in distinct ways over and above the global factor, instead of treating need satisfaction as a monolithic construct. Thus, the present study proposes:

*H2*: *BPNS is positively related to TBB*.

Work engagement provides a complementary motivational pathway to TBB. Engaged employees tend to invest discretionary effort and display extra-role behaviour (Mazzetti et al., 2021; Sulea et al., 2012). Because TBB are discretionary, team-directed behaviours, engaged employees may be especially likely to enact mood-enhancing, energising, and unifying actions that shape the team climate (Fortuin et al., 2021; Fortuin et al., 2023). The components of engagement map onto this expectation in specific ways: vigour supplies the energetic capacity for energising acts, dedication reflects an identification with the work that makes investment in the team meaningful, and absorption indicates an involvement that may spill over into enthusiasm during team interactions (Bakker, 2022; Mazzetti et al., 2021). This is consistent with emotional contagion processes whereby affective states and behavioural cues can spread within teams and influence shared mood (Bakker, 2022; Herrando and Constantinides, 2021). Accordingly:

*H3*: *Work engagement is positively related to TBB*.

Taken together, BPNS is expected to relate to work engagement, and engagement may represent the motivational state through which need satisfaction translates into team-directed discretionary behaviour. In other words, need-satisfied employees may be more engaged, and that engagement may increase the likelihood of initiating behaviours that boost team climate, positioning work engagement as a mediator in these relationships. This expectation is consistent with theorising that positions engagement as an expression of fulfilled psychological needs (Pincus, 2023) and with recent work framing team emergent states as expressions of members’ emotional needs (Pincus, 2025). It also follows broaden-and-build reasoning, in which positive motivational states widen momentary thought-action repertoires and make discretionary, team-directed behaviour more likely (Fredrickson, 2013). Therefore:

*H4*: *Work engagement is a mediator in the relation between BPNS and TBB*.

All in all, this study investigates whether team-boosting behaviours manifest within environments where employees’ basic psychological needs are satisfied, creating positive spillover within teams through employees’ engagement at work.

## 3 Method

A cross-sectional non-experimental survey design was employed and is effective and suitable for gathering data from a large sample with limited research resources (Spector, 2019). Cross-sectional research is suitable for conceptualising the direct and indirect relations among the constructs in a mediation model, although it cannot establish the temporal or causal ordering of these relations (Maxwell and Cole, 2007). Primary data were collected and the participants utilised self-report surveys.

### 3.1 Participants and procedure

The analytic sample consisted of 332 employees who provided usable responses on the study variables, and all models were estimated on this sample. Demographic frequencies are reported against the valid responses per demographic item, as not all participants answered every demographic question. Most of the participants were female employees (*n* = 200; 63.49%), and the group consisted mostly of African (*n* = 159; 50.48%) and white (*n* = 110; 34.92%) participants. Participants were from a broad range of sectors, but a plurality of the participants were in the financial and business services sector (*n* = 88; 28.02%), followed by the participants in other sectors (*n* = 33; 10.51%) and the retail, motor trade and commercial agents and allied services sector (*n* = 24; 7.64%). Participants’ presence at work was mainly full-time at work (*n* = 260; 82.54%). Lastly, most participants were in teams with more than 25 members (*n* = 78; 24.76%), followed by a team of 6–10 members (*n* = 74; 23.76%), and then teams of 2–5 members (*n* = 60; 19.05%). Respondents answered the team-referenced survey items with reference to their own current work team (‘our team’). Consistent with the anonymous design approved for this study, no team or organisational identifiers were collected, and all data were therefore collected and modelled at the individual level.

Ethics clearance was obtained (Ref: [removed for peer-review]), after which participants were recruited through a non-probability approach, with the survey distributed on online platforms and through participating organisations, using an information letter detailing purpose, consent, confidentiality, and data protection. Surveys were administered in self-complete hard-copy and online formats, with secure collection and storage procedures. Because the online survey link was distributed openly, an exact response rate could not be determined. Participation was voluntary and anonymous, data was used solely for this study and stored securely, and aggregated findings will be disseminated publicly, with optional individual feedback provided to interested participants.

### 3.2 Measuring instruments

*Need satisfaction*: The scale utilised for deriving data regarding participants’ satisfaction was the *Basic Psychological Need Satisfaction and Frustration—Work Domain (BPNFS)* (Chen et al., 2015). We used the 12 survey items to assess BPNS at work. This scale focuses on measuring the three psychological needs: autonomy satisfaction (e.g., ‘At work, I feel a sense of choice and freedom in the things I undertake’), relatedness satisfaction (e.g., ‘I feel that the people I care about at work also care about me’), and competence satisfaction (e.g., ‘I feel confident that I can do things well on my job’) (Chen et al., 2015). This measure is answered on a 7-point Likert scale, ranging from ‘strongly disagree’ (1) to ‘strongly agree’ (7), in which each respective construct has three allocated items. This scale reflects a higher internal reliability and factor structure relating to work-related contexts (Chen et al., 2015). This scale is cross-culturally validated with reliability coefficients larger than 0.70 (including South Africa) and also supported by other studies (Cordeiro et al., 2016; Costa et al., 2018; Schultz et al., 2015). In the present sample, the McDonald’s omega coefficient for the global need satisfaction factor was 0.94.

*Work engagement*: The short version of the Utrecht Work Engagement Scale (UWES-9) was used to measure work engagement (Schaufeli et al., 2006). This 9-item scale measures the following characteristics: vigour (e.g., ‘At my work, I feel bursting with energy’), dedication (e.g., ‘I am enthusiastic about my job’) and absorption (e.g., ‘I am immersed in my work’) on a 7-point Likert scale ranging from ‘never’ (0) to ‘always’ (6) (Schaufeli et al., 2006). Within workplace research, this scale is well known for measuring work engagement across borders with appropriate reliability coefficients above 0.70 (Breaugh, 2021; Costa et al., 2014; Kulikowski, 2017). The omega coefficient for work engagement in this study was 0.95.

*Team-boosting behaviours (TBB)*: The 18-item self-report scale was used to measure TBB in the study (Fortuin et al., 2021). This scale is answered on a 7-point Likert scale ranging from ‘never’ (0) to ‘always’ (6) and includes mood-enhancing behaviours (e.g., ‘I make sure that there is laughter in our team’), energising behaviours (e.g., ‘I take initial action to set our team in motion’) and uniting behaviours (e.g., ‘I involve all my teammates in what we do’). The scale has shown satisfactory reliability and criterion validity with team variables such as team proactivity, group cohesion and positive team mood (Bakker, 2022; Fortuin et al., 2021). The TBB scale reflected satisfactory reliability coefficients: mood-enhancing behaviours (*α* = 0.92), energising behaviours (*α* = 0.91), and uniting behaviours (*α* = 0.88) (Fortuin et al., 2021). These coefficients stem from the original validation study; internal consistency estimates for all scales in the present sample were computed as McDonald’s omega coefficients from the final measurement model and are reported in the Results and in Supplementary Table 1. In the present sample, the omega coefficients were 0.94 for mood-enhancing behaviours, 0.95 for energising behaviours, and 0.91 for unifying behaviours.

### 3.3 Statistical analyses

Need satisfaction, work engagement and team-boosting behaviours were considered latent variables and were situated within a mediation model that required structural equation modelling methods (SEM) to test the model, first through confirmatory factor analysis (Brown, 2015; Wang and Wang, 2020). Mplus 8.8 (Muthén and Muthén, 2022) was used to analyse the conceptual research model proposed in this study. Five confirmatory factor analytic measurement models were tested: (1) a model where need satisfaction was specified as a first-order unidimensional factor with the remaining factors (work engagement, mood-enhancing, energising, unifying) as first-order factors; (2) a model which included all factors as first-order factors; (3) a bifactor confirmatory factor analysis model of need satisfaction (global factor + three factors with no cross-loadings allowed) with the remaining factors as first-order specifications as in model 1; (4) an exploratory structural equation modelling (ESEM) for need satisfaction without a global factor with the remaining factors as specified in model 1; and (5) a bifactor exploratory structural equation modelling (BESEM) for need satisfaction (global factor + three specific factors with cross-loading specifications) within a model that contains the remaining factors as first-order specifications as in model 1. The benefit of the ESEM technique is that, due to allowing for approximately zero cross-loadings, wording and context effects are more controlled for across the factors. The mean- and variance-adjusted weighted least squares estimator (WLSMV) was used to estimate the model.

To examine the fit of these models mentioned above, the comparative fit index (CFI ≥ 0.90), Tucker-Lewis index (TLI ≥ 0.90), root mean square error of approximation (RMSEA <0.08), and standardised root mean residual (SRMR <0.08) were used. McDonald’s omega coefficients (ω) were used to determine the internal consistency of first-order model factors, with values of approximately 0.70 and above deemed acceptable. However, in the case of BESEM models, research has shown that omega coefficients of even as low as 0.50 may indicate meaningful specific factors (Perreira et al., 2018).

Furthermore, a correlation matrix was generated that contains relationships between the variables from the CFA and ESEM models. The effect sizes for correlations were considered in line with normal conventions: small (*r* ≥ 0.10), medium (*r* ≥ 0.30), or large (*r* ≥ 0.50). Finally, a structural model was specified that included all meaningful hypothesised paths. Bootstrapping was used as the preferred method to generate 95% confidence intervals for the potential indirect effects in the model that should not cross zero to remain meaningful.

Given the single-source self-report design, common method variance (CMV) was considered through procedural and statistical means (Podsakoff et al., 2012). Procedurally, anonymity and voluntary participation is suggested to reduce evaluation apprehension. The predictor and outcome measures also used different response anchors (agreement-based anchors for BPNS; frequency-based anchors for work engagement and TBB), which reduces anchor-driven covariation. Statistically, no marker variable was included in the model. However, the measurement model comparisons reported in Table 1 supported a multidimensional structure over more constrained alternatives, which is difficult to reconcile with a single method factor accounting for the observed covariation. The potential for CMV to have inflated the associations is acknowledged in the limitations.

**Table 1:** Fit statistics of the measurement models.

Description^*^	χ^2^	df	CFI	TLI	RMSEA [90% CI]	SRMR
Model 1	1877.057	692	0.957	0.954	0.074 [0.070, 0.078]	0.056
Model 2	1531.954	681	0.969	0.967	0.063 [0.059, 0.067]	0.046
Model 3	1403.660	668	0.974	0.971	0.059 [0.055, 0.064]	0.042
Model 4	1390.633	663	0.974	0.971	0.059 [0.055, 0.063]	0.041
Model 5	1320.608	656	0.976	0.973	0.057 [0.052, 0.061]	0.041

* = For a description of each model see the statistical analyses section earlier; χ^2^ = Chi-square; df = Degrees of freedom; CFI = Comparative Fit Index; TLI = Tucker-Lewis Index; RMSEA = Root Mean Square Error of Approximation; SRMR = Standardised Root Mean Squared Residual.

## 4 Results

### 4.1 Measurement models

The results of the latent variable modelling for the five specified measurement models are provided in Table 1.

All the models fit the data well. However, there were minor improvements as the models progressed to the bifactor-ESEM model. The ESEM approach is also justified by considering the correlations (see Table 2), which indicated that the correlations were reduced in the ESEM approach (model 4) compared to the CFA approach (model 2). The addition of a global factor of need satisfaction is also in line with the literature and, given the improvements in model fit, it was decided to accept this model for the study (*χ*^2^ = 1320.61; df = 656; CFI = 0.976; TLI = 0.973; RMSEA = 0.057 [0.052, 0.061]; SRMR = 0.041).

**Table 2:** Correlation matrix for the latent variables.

Variables	1	2	3	4	5	6	7
1. Autonomy satisfaction	–	0.33a	0.43a	0.80b	0.39a	0.52b	0.49a
2. Competence satisfaction	0.79b	–	0.20	0.39a	0.43a	0.63b	0.62b
3. Relatedness satisfaction	0.79b	0.59b	–	0.39a	0.49a	0.37a	0.45a
4. Work engagement	0.80b	0.61b	0.53b	–	0.56b	0.64b	0.56b
5. Mood enhancing behaviours	0.55b	0.51b	0.54b	0.56b	–	0.74b	0.73b
6. Energising behaviours	0.68b	0.68b	0.48a	0.64b	0.74b	–	0.88b
7. Unifying behaviours	0.68b	0.65b	0.55b	0.60b	0.73b	0.88b	–

CFA correlations below the diagonal; ESEM correlations above the diagonal; all correlations *p* < 0.01; *a* = Medium effect; *b* = Large effect.

### 4.2 Correlation matrices for the CFA and ESEM models

The correlation matrices for model 2 and model 4 are provided in Table 2.

The correlations indicate that the ESEM-within-CFA model (above the diagonal) reduced the size of the correlation between the need satisfaction components compared to the CFA (below the diagonal). The average correlation between the components was 0.72 (large effect) in the CFA versus 0.32 (medium effect) in the ESEM model. This provided support for the relaxation of the exactly-zero cross-loadings of CFA models. However, the large correlations between need satisfaction factors in CFA models also indicate an underlying global factor that may not be accounted for—as indeed found in the evidence above for the BESEM-within-CFA model, which forms the foundation of these results.

All the omega coefficients were acceptable (*ω* > 0.70) for the global BPNS, work engagement and TBB. However, only the competence and relatedness specific factors were above (*ω* > 0.50). Therefore, even though the autonomy specific factor was still modelled in the measurement model, we excluded its paths from the path model. See Supplementary Table 1 for the factor loadings and omega coefficients of the final measurement model used in the analyses.

### 4.3 Structural model

The fit of the structural model was highly acceptable (*χ*^2^ = 1307.07; df = 660; CFI = 0.977; TLI = 0.974; RMSEA = 0.056 [0.051, 0.060]; SRMR = 0.042). In the structural model, global need satisfaction was used as independent variable, as well as the two remaining specific factors according to the omega coefficients mentioned earlier (competence and relatedness). Path results are given in Table 3.

**Table 3:** Direct path results for the structural model.

Structural path	*β*	SE	*p*	Result
Global: need satisfaction → Work engagement	0.76^*^	0.04	<0.001	Significant
Specific: competence → Work engagement	−0.16	0.11	0.148	Not significant
Specific: relatedness → Work engagement	−0.15	0.12	0.223	Not significant
Global: need satisfaction → Mood enhancing behaviours	0.21^*^	0.10	0.039	Significant
Specific: competence → Mood enhancing behaviours	0.20^*^	0.09	0.034	Significant
Specific: relatedness → Mood enhancing behaviours	0.23^*^	0.08	0.006	Significant
Work engagement → Mood enhancing behaviours	0.47^*^	0.11	<0.001	Significant
Global: need satisfaction → Energising behaviours	0.41^*^	0.10	<0.001	Significant
Specific: competence → Energising behaviours	0.29^*^	0.07	<0.001	Significant
Specific: relatedness → Energising behaviours	−0.04	0.08	0.613	Not significant
Work engagement → Energising behaviours	0.37^*^	0.11	0.010	Significant
Global: need satisfaction → Unifying behaviours	0.46^*^	0.11	<0.001	Significant
Specific: competence → Unifying behaviours	0.29^*^	0.08	<0.001	Significant
Specific: relatedness → Unifying behaviours	0.06	0.08	0.464	Not significant
Work engagement → Unifying behaviours	0.30	0.12	0.010	Significant

*β* = standardised beta coefficient; SE = Standard error; *p* = Two-tailed statistical significance; ^*^ = *p* < 0.05.

The global BPNS had significant path results to work engagement (*β* = 0.76, SE = 0.02; *p* < 0.001), mood enhancing behaviours (*β* = 0.21, SE = 0.10; *p* = 0.039), energising behaviours (*β* = 0.41, SE = 0.10; *p* < 0.001), and unifying behaviours (*β* = 0.46, SE = 0.11; *p* < 0.001). Of the specific factors, competence satisfaction had three significant paths to mood enhancing behaviours (*β* = 0.20, SE = 0.09; *p* = 0.034), energising behaviours (*β* = 0.29, SE = 0.07; *p* < 0.001), and unifying behaviours (*β* = 0.29, SE = 0.08; *p* < 0.001)—but a non-significant path to work engagement (*p* = 0.148). Furthermore, specific relatedness satisfaction only had positive significant paths to mood enhancing behaviours (*β* = 0.23, SE = 0.08; *p* = 0.006). Finally, it should be noted that work engagement had significant paths to all three team-boosting behaviours: mood enhancing (*β* = 0.47, SE = 0.11; *p* < 0.001), energising (*β* = 0.37, SE = 0.11; *p* = 0.010), and unifying (*β* = 0.30, SE = 0.12; *p* = 0.010).

Figure 1 presents all the significant path results with explained variances included for the dependent variables.

**Figure 1:**
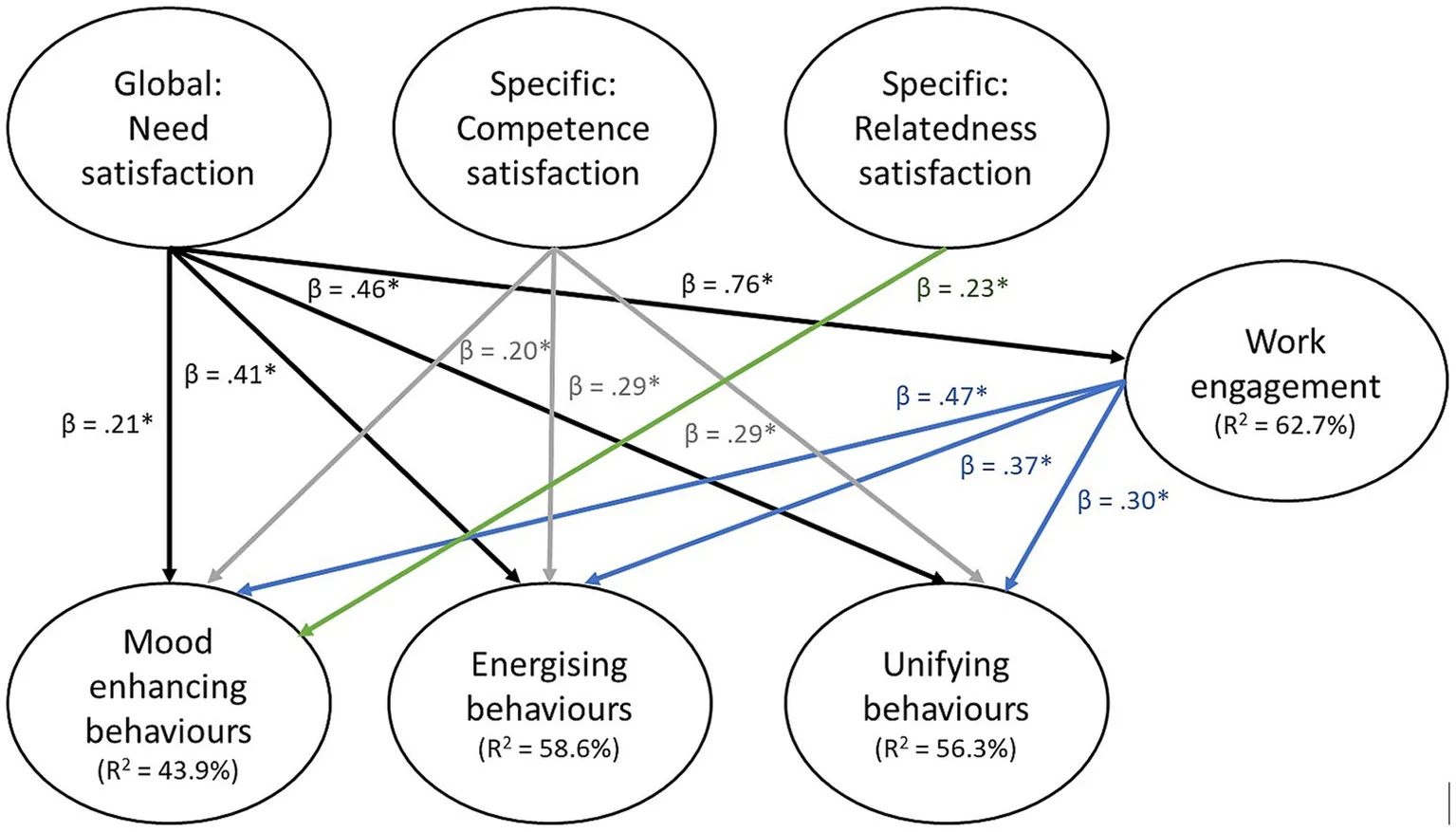
Significant path results. Source: Authors’ own work.

### 4.4 Indirect effects

Table 4 provides the bootstrapped indirect effects from the model, with lower and upper 95% confidence intervals.

**Table 4:** Indirect paths for the structural model.

Indirect path	*β*	L95% CI	U95% CI
Global: need satisfaction → WE → Mood enhancing behaviours	0.36^*^	0.20	0.54
Global: need satisfaction → WE → Energising behaviours	0.28^*^	0.14	0.45
Global: need satisfaction → WE → Unifying behaviours	0.23^*^	0.07	0.42
Specific: competence → WE → Mood enhancing behaviours	−0.07	−0.23	0.02
Specific: competence → WE → Energising behaviours	−0.06	−0.22	0.01
Specific: competence → WE → Unifying behaviours	−0.05	−0.20	0.01
Specific: relatedness → WE → Mood enhancing behaviours	−0.07	−0.21	0.06
Specific: relatedness → WE → Energising behaviours	−0.06	−0.14	0.05
Specific: relatedness → WE → Unifying behaviours	−0.04	−0.13	0.04

*β* = standardised value; WE = work engagement; ^*^ = does not include zero; L 95% CI = lower 95% confidence interval; U 95% CI = upper 95% confidence interval; Source: Authors’ own work.

Interestingly, only the global BPNS factor had a meaningful indirect relation to all the team-boosting behaviours through work engagement (as a mediating variable). The specific factors’ 95% confidence intervals all included zero and were therefore not meaningful for further consideration. The only evidence for potential mediation thus was work engagement as a mediator between global BPNS and team-boosting behaviours. Considering that all the direct effects were also significant, this is evidence for complementary mediation (Zhao et al., 2010).

## 5 Discussion

This study examined direct and indirect relationships between basic psychological need satisfaction (BPNS), work engagement, and team-boosting behaviours (TBB) in a diverse sample of employees. To our knowledge, no studies have yet integrated BPNS, work engagement, and team-boosting behaviours within a single model. Extending SDT-informed work motivation research into the team domain, the findings suggest that employees’ BPNS is associated with higher work engagement and, in turn, more frequent discretionary behaviours that can lift the team’s mood, energy, and cohesion. In this way, the study highlights a plausible bottom-up pathway through which individual motivational experiences may translate into interpersonal behaviours that strengthen the social climate of teams (Ryan and Deci, 2017; Van den Broeck et al., 2016; Vansteenkiste et al., 2020).

A notable finding was that BPNS was best represented by a bifactor-ESEM structure comprising a strong global BPNS factor and weaker specific factors. In this model, autonomy satisfaction did not retain sufficient specificity once the global factor was modelled, suggesting that autonomy satisfaction may have been largely embedded in the broader experience of overall need satisfaction. Substantively, this aligns with SDT’s view of need satisfaction as a set of interdependent “psychological nutrients” that jointly support functioning (Ryan and Deci, 2017; Vansteenkiste et al., 2020).

Consistent with Hypothesis 1, global BPNS showed a strong positive association with work engagement. This accords with prior work indicating that need satisfaction is an important antecedent and/or mechanism through which supportive work conditions relate to engagement (Breaugh, 2021; Heyns et al., 2022; Morin et al., 2023; Rahmadani et al., 2019; Vansteenkiste et al., 2020). Employees who feel volitional in their actions (autonomy), capable of meeting demands (competence), and connected to others (relatedness) may be more likely to experience engagement as a fulfilling work-related state (Ryan and Deci, 2017; Van den Broeck et al., 2016; Ryan et al., 2022). In contrast, competence satisfaction did not show a unique association with work engagement in the presence of the modelled global BPNS factor. Because the competence satisfaction items are also represented in the global BPNS factor, the competence-specific factor reflects variance in competence satisfaction over and above employees’ general level of BPNS. Thus, the results suggest that engagement was primarily associated with the shared, integrated component of need satisfaction rather than competence-specific variance beyond global BPNS. This diverges from findings in more occupationally specific contexts (e.g., nurses), where competence may be especially salient for work engagement (Liebenberg et al., 2022). One interpretation is that, in a heterogeneous sample spanning roles and industries, engagement is driven more strongly by the integrated experience of overall BPNS than by any single need dimension in isolation (Vansteenkiste et al., 2020).

Supporting Hypothesis 2, global BPNS was positively associated with TBB. This is consistent with the SDT proposition that satisfied needs energise growth and adaptive functioning and guide behaviour in social environments (Ryan and Deci, 2017; Van den Broeck et al., 2016; Vansteenkiste et al., 2020). When employees experience need fulfilment, they may have greater capacity to attend to the team’s socio-emotional environment—using humour or positive reframing to improve the atmosphere (mood-enhancing), initiating momentum and collaborative action (energising), and strengthening inclusion and cohesion (unifying). Beyond this shared BPNS component, the competence-specific factor—reflecting competence satisfaction over and above employees’ overall level of need fulfilment—was positively associated with all three TBB dimensions. This suggests that, holding global BPNS constant, employees who feel relatively more effective may be more likely to initiate and persist in behaviours that enhance team functioning. Additionally, the relatedness-specific factor was positively associated with mood-enhancing behaviours, which is theoretically coherent given that feeling connected to others is closely tied to affiliative and prosocial expression (Chen et al., 2015; Tao et al., 2018). This pattern aligns with prior work conceptualising relatedness as especially relevant to interpersonal warmth and connection, which may be expressed through humour, cheerfulness, and sharing that lifts the team’s affective tone (Chen et al., 2015; Fortuin et al., 2021). This reading is consistent with the group affect literature, in which member behaviours act as inputs to affective convergence within teams (Barsade & Knight, 2015), and with work on team emotional intelligence norms that govern how teams attend to members’ emotions and needs (Druskat & Wolff, 2001).

In line with Hypothesis 3, work engagement was positively associated with all three TBB dimensions. This complements evidence linking engagement to extra-role and citizenship behaviours by suggesting that engagement may also be expressed through team-directed acts that shape the team’s affective and relational context (Wörtler et al., 2020). While prior work has examined how TBBs may relate to outcomes such as performance via teamwork engagement (Fortuin et al., 2023), the present findings support the reverse pathway: an engaged individual appears more likely to enact team-boosting behaviours. This interpretation is consistent with frameworks in which individual engagement contributes as an input to team engagement and related team processes (e.g., Costa et al., 2014).

Finally, Hypothesis 4 was also supported: work engagement was a mediator in the relationship between global BPNS and all three TBB dimensions. Importantly, meaningful indirect effects emerged only for global BPNS rather than for any specific factors, suggesting that a global integrated experience of need fulfilment may be especially important for fostering work engagement, which then relates to TBB. This is consistent with evidence that need satisfaction and engagement jointly support discretionary, other-oriented behaviours in work contexts (Wörtler et al., 2020).

### 5.1 Practical implications

This study contributes to the literature by clarifying how employees’ BPNS relates to work engagement and TBB. Given the cross-sectional, single-source design, the implications that follow should be read as tentative directions that assume replication in stronger designs, not as established intervention effects. Practically, the findings suggest that organisations should prioritise work environments that support employees’ overall need satisfaction/fulfilment (autonomy, competence, and relatedness). Global need satisfaction showed a strong positive association with work engagement and was also positively associated with all three TBB facets (mood-enhancing, energising, and unifying). In addition, work engagement was positively associated with each TBB facet, and global need satisfaction showed meaningful indirect associations with TBB through work engagement. Together, these results imply that a broadly need-supportive climate may both energise employees’ engagement and promote constructive interpersonal behaviour within teams.

At the same time, the pattern of results points to specific, actionable emphasis areas. Beyond overall need fulfilment, competence-related experiences were positively associated with all three TBB facets. This suggests that organisations may be able to strengthen team functioning by supporting employees’ sense of effectiveness; helping them feel capable of initiating action, sustaining momentum, and contributing to cohesion. In practice, this can be promoted through clear role expectations, timely performance feedback, coaching, and access to resources and training that enable employees to succeed in their tasks. It may also be supported by job and team design practices that create conditions for mastery experiences (e.g., manageable challenge, opportunities to use skills, and visible progress in work).

Relatedness-related experiences also appear relevant, particularly for behaviours that lift the team’s affective tone. Beyond global BPNS, relatedness was positively associated with mood-enhancing behaviours, suggesting that employees who feel more connected may be more likely to use humour, positivity, and supportive communication that improve the team’s socio-emotional climate. Organisations can facilitate this by strengthening psychological safety, respectful interaction norms, and opportunities for constructive collaboration, including team routines that promote inclusion and interpersonal connection (e.g., structured check-ins, peer support, and onboarding practices that build belonging).

Consistent with Matosic et al. (2016), need-supportive environments often involve social support from colleagues and managers, which can foster more self-determined motivation. When employees experience support through clear communication channels and constructive collaboration, it may nurture relatedness and strengthen team bonds (Jungert et al., 2018). Moreover, autonomy support may be particularly important in diverse contexts because it acknowledges employees’ perspectives and provides choice and rationale—practices that can help individuals feel respected and volitional in their work (Ryan and Deci, 2020). Accordingly, organisations can implement interventions that help leaders and teams develop need-supportive communication (e.g., offering meaningful choice where possible, explaining constraints, inviting input, and recognising competence through informational feedback).

Finally, need-crafting approaches may offer a practical route for enhancing overall need fulfilment at both individual and team levels (Mokgata et al., 2023; Putter et al., 2024). Such interventions can help employees assess their work tasks and team processes, identify opportunities to craft for greater autonomy, competence, and relatedness, and develop concrete plans for implementation. Teams may benefit from creating a shared “need inventory” that identifies which practices support need fulfilment (e.g., how the team shares information, coordinates work, provides feedback, and includes members), thereby translating need-support principles into everyday routines.

### 5.2 Limitations and directions for future research

First, although a sufficient sample was collected among employees in South Africa this inevitably limits generalizability. Accordingly, findings should be interpreted cautiously, and replication in other countries, specific sectors or occupational groups is recommended to validate the model and reduce potential sampling bias, including self-selection related to voluntary online recruitment. Second, while a cross-sectional, self-report design was appropriate for efficient data collection from a large sample, it restricts causal inference. Cross-sectional estimates of mediation can be biased relative to longitudinal estimates (Maxwell and Cole, 2007), and reverse or reciprocal orderings, such as engagement colouring perceived need satisfaction or enacted TBB building relatedness, cannot be ruled out. The single-source design also means that common method variance may have inflated the observed associations (Podsakoff et al., 2012). This concern applies most directly to TBB, which were self-rated; ratings of one’s own team-boosting behaviour are vulnerable to social desirability, and future studies should collect peer- or team-rated TBB, for example through round-robin ratings within identified teams. Future longitudinal or experimental studies are therefore needed to examine changes in need satisfaction, team climate, and TBB over time and to evaluate targeted interventions. Third, although a variable-centered approach allowed for parsimonious model testing, individual differences were not considered. Adopting person-centered approaches and incorporating personality measures in future research may yield more nuanced TBB profiles. Fourth, accurate interpretation of specific factors in bifactor-ESEM models can be challenging beyond the global factor and any interpretation of the specific factors should therefore be considered with caution. For example, the competence specific factor may reflect the residual capturing of competence-specific nuances (e.g., effectiveness-related perceptions or role/task specificity) rather than a distinct ‘non-work’ competence construct. Fortunately, our results for global BPNS were robust. Relatedly, some latent correlations were large (e.g., between the need satisfaction components and work engagement, and between energising and unifying behaviours); the ESEM specification reduced these correlations relative to CFA (see Table 2), and the bifactor structure separates shared from specific variance. Even so, such correlations may warrant some caution when interpreting the distinctness of adjacent constructs and the stability of the structural estimates. Fifth, although TBB are directed at the team, all constructs were measured and modelled at the individual level, with no team identifiers available to model nesting. The findings therefore speak to individual, team-directed behaviour, not to team-level climate; multilevel designs with identified teams are needed to examine compositional and emergent effects. Sixth, emotional contagion and broaden-and-build theory served as interpretive framing, not as tested mechanisms, as positive affect was not measured; future studies could include affect variables to test these pathways directly. Seventh, the operationalisation of need satisfaction was bounded to the three needs specified by SDT, as measured with the validated work-domain scale; candidate needs such as beneficence (Martela and Ryan, 2016) and novelty (González-Cutre et al., 2016) were therefore not measured. The evidence to date, however, suggests that beneficence functions as a wellness enhancer rather than a basic psychological need, because its frustration adds no unique variance in ill-being once frustration of the three needs is accounted for (Martela and Ryan, 2020; Vansteenkiste et al., 2020; Vansteenkiste et al., 2023). Given the conceptual proximity between beneficence and TBB, future research could examine whether a sense of positive impact operates as an antecedent, correlate, or outcome of these behaviours. Finally, data were collected during a period of evolving work arrangements (post-COVID-19); consequently, future research should examine potential differences between remote, onsite, and hybrid contexts to account for contextual influences.

## 6 Conclusion

This study found preliminary evidence that employees’ global basic psychological need satisfaction was positively associated with work engagement and, directly and indirectly, with team-boosting behaviours. By supporting a model in which need satisfaction relates to work engagement, which in turn relates to mood-enhancing, energising, and unifying team behaviours, the findings point to the role that psychological need fulfilment may play in shaping positive team climates.

## Data Availability

The raw data supporting the conclusions of this article will be made available by the authors, without undue reservation.
