# Supplementary material for: Satisfied employees, stronger teams: linking need satisfaction to team-boosting behaviours via work engagement
Source: Front Psychol. 2026 Sep 23;17:1917631. doi: 10.3389/fpsyg.2026.1917631 (PMC13642122; doi:10.3389/fpsyg.2026.1917631)
Supplement: Supplementary file 1 [file Table_1.DOCX]

**Supplementary Material**

***Factor loadings and omega coefficients of the final measurement model***

The factor loadings for the measurement model (Model 5) are presented in Supplementary Table 1 below.

**Supplementary Table 1. Standardised loadings and omega coefficients for the latent factors of the final measurement model**

| Factor | Item | Loading | SE | p | ω |
| --- | --- | --- | --- | --- | --- |
| Work engagement (WE) | ENG1 | 0.82 | 0.03 | < .001 | 0.95 |
|  | ENG2 | 0.87 | 0.02 | < .001 |  |
|  | ENG3 | 0.84 | 0.02 | < .001 |  |
|  | ENG4 | 0.92 | 0.02 | < .001 |  |
|  | ENG5 | 0.91 | 0.02 | < .001 |  |
|  | ENG6 | 0.86 | 0.02 | < .001 |  |
|  | ENG7 | 0.82 | 0.02 | < .001 |  |
|  | ENG8 | 0.73 | 0.03 | < .001 |  |
|  | ENG9 | 0.65 | 0.04 | < .001 |  |
| Global: Need satisfaction | **AUTOS1** | 0.67 | 0.04 | < .001 | 0.94 |
|  | **AUTOS2** | 0.63 | 0.01 | < .001 |  |
|  | **AUTOS3** | 0.70 | 0.08 | < .001 |  |
|  | **AUTOS4** | 0.80 | 0.04 | < .001 |  |
|  | **COMPS1** | 0.57 | 0.05 | < .001 |  |
|  | **COMPS2** | 0.51 | 0.02 | < .001 |  |
|  | **COMPS3** | 0.76 | 0.04 | < .001 |  |
|  | **COMPS4** | 0.50 | 0.05 | < .001 |  |
|  | **RELAS1** | 0.61 | 0.04 | < .001 |  |
|  | **RELAS2** | 0.57 | 0.05 | < .001 |  |
|  | **RELAS3** | 0.62 | 0.06 | < .001 |  |
|  | **RELAS4** | 0.69 | 0.05 | < .001 |  |
| Specific: Autonomy satisfaction | **AUTOS1** | -0.03 | 0.09 | 0.697 | 0.35 |
|  | **AUTOS2** | 0.19 | 0.08 | 0.026 |  |
|  | **AUTOS3** | 0.71 | 0.08 | 0.000 |  |
|  | **AUTOS4** | -0.10 | 0.10 | 0.345 |  |
|  | *COMPS1* | 0.05 | 0.07 | 0.516 |  |
|  | *COMPS2* | 0.02 | 0.00 | 0.000 |  |
|  | *COMPS3* | 0.05 | 0.09 | 0.596 |  |
|  | *COMPS4* | 0.01 | 0.08 | 0.892 |  |
|  | *RELAS1* | -0.04 | 0.00 | 0.000 |  |
|  | *RELAS2* | 0.18 | 0.07 | 0.010 |  |
|  | *RELAS3* | 0.06 | 0.07 | 0.391 |  |
|  | *RELAS4* | 0.04 | 0.08 | 0.629 |  |
| Specific: Competence satisfaction | **COMPS1** | 0.52 | 0.06 | 0.000 | 0.52 |
|  | **COMPS2** | 0.53 | 0.06 | 0.000 |  |
|  | **COMPS3** | 0.20 | 0.07 | 0.004 |  |
|  | **COMPS4** | 0.43 | 0.05 | 0.000 |  |
|  | *AUTOS1* | -0.08 | 0.07 | 0.242 |  |
|  | *AUTOS2* | 0.14 | 0.00 | 0.000 |  |
|  | *AUTOS3* | -0.05 | 0.01 | 0.000 |  |
|  | *AUTOS4* | -0.27 | 0.08 | 0.001 |  |
|  | *RELAS1* | -0.01 | 0.00 | 0.000 |  |
|  | *RELAS2* | -0.01 | 0.06 | 0.859 |  |
|  | *RELAS3* | 0.05 | 0.07 | 0.489 |  |
|  | *RELAS4* | -0.07 | 0.07 | 0.363 |  |
| Specific: Relatedness satisfaction | **RELAS1** | 0.51 | 0.09 | 0.000 | 0.65 |
|  | **RELAS2** | 0.55 | 0.05 | 0.000 |  |
|  | **RELAS3** | 0.52 | 0.05 | 0.000 |  |
|  | **RELAS4** | 0.48 | 0.06 | 0.000 |  |
|  | *AUTOS1* | 0.11 | 0.08 | 0.164 |  |
|  | *AUTOS2* | -0.13 | 0.00 | 0.000 |  |
|  | *AUTOS3* | 0.03 | 0.00 | 0.000 |  |
|  | *AUTOS4* | -0.05 | 0.09 | 0.570 |  |
|  | *COMPS1* | 0.06 | 0.06 | 0.315 |  |
|  | *COMPS2* | 0.01 | 0.00 | 0.000 |  |
|  | *COMPS3* | -0.10 | 0.06 | 0.090 |  |
|  | *COMPS4* | -0.05 | 0.06 | 0.439 |  |
| Mood enhancing behaviours | MOOD1 | 0.88 | 0.02 | < .001 | 0.94 |
|  | MOOD2 | 0.83 | 0.02 | < .001 |  |
|  | MOOD3 | 0.88 | 0.02 | < .001 |  |
|  | MOOD4 | 0.91 | 0.02 | < .001 |  |
|  | MOOD5 | 0.87 | 0.02 | < .001 |  |
|  | MOOD6 | 0.71 | 0.03 | < .001 |  |
| Energising behaviours | ENERGY1 | 0.88 | 0.02 | < .001 | 0.95 |
|  | ENERGY2 | 0.83 | 0.02 | < .001 |  |
|  | ENERGY3 | 0.88 | 0.02 | < .001 |  |
|  | ENERGY4 | 0.84 | 0.02 | < .001 |  |
|  | ENERGY5 | 0.94 | 0.01 | < .001 |  |
|  | ENERGY6 | 0.88 | 0.02 | < .001 |  |
| Unifying behaviours | UNITY1 | 0.95 | 0.01 | < .001 | 0.91 |
|  | UNITY2 | 0.96 | 0.01 | < .001 |  |
|  | UNITY3 | 0.79 | 0.02 | < .001 |  |
|  | UNITY4 | 0.49 | 0.04 | < .001 |  |
|  | UNITY5 | 0.68 | 0.03 | < .001 |  |
|  | UNITY6 | 0.84 | 0.02 | < .001 |  |

*Notes:* SE = standard error; ω = omega reliability coefficient; bolded text = target loadings of the bifactor ESEM model; italics = cross-loadings of the bifactor ESEM model; *p* (0.000) < 0.001.

As seen from the factor loadings, the global need satisfaction factor was well-defined, with all loadings being at least 0.50, with the highest loading reaching 0.80 (M_λ_ = 0.64). The omega coefficient for global need satisfaction was also highly acceptable (ω = 0.93). For the specific factors, autonomy satisfaction did not perform well (ω = 0.44) in the presence of the global factor, two target items were not significant, and only one of the two remaining items had a near acceptable loading (M_λ_ = 0.26), but competence (ω = 0.61) and relatedness (ω = 0.76) satisfaction were acceptable. This indicated that the experience of autonomy satisfaction was most likely already taken up in the global need satisfaction score. Furthermore, all the remaining factors in the model showed highly acceptable according to the loadings and omega coefficients presented.
